# A systematic evaluation of normalization methods in quantitative label-free proteomics

**DOI:** 10.1093/bib/bbw095

**Published:** 2016-10-02

**Authors:** Tommi Välikangas, Tomi Suomi, Laura L Elo

**Affiliations:** 1 Computational Biomedicine Group at the Turku Centre for Biotechnology Finland; 2 Computational Biomedicine research group at the Turku Centre for Biotechnology Finland; 3 Computational Biomedicine at Turku Centre for Biotechnology, University of Turku, Finland

**Keywords:** proteomics, normalization, label free, bias, differential expression, logarithmic fold change, quantitation, intragroup variation, reproducibility, mass spectrometry

## Abstract

To date, mass spectrometry (MS) data remain inherently biased as a result of reasons ranging from sample handling to differences caused by the instrumentation. Normalization is the process that aims to account for the bias and make samples more comparable. The selection of a proper normalization method is a pivotal task for the reliability of the downstream analysis and results. Many normalization methods commonly used in proteomics have been adapted from the DNA microarray techniques. Previous studies comparing normalization methods in proteomics have focused mainly on intragroup variation. In this study, several popular and widely used normalization methods representing different strategies in normalization are evaluated using three spike-in and one experimental mouse label-free proteomic data sets. The normalization methods are evaluated in terms of their ability to reduce variation between technical replicates, their effect on differential expression analysis and their effect on the estimation of logarithmic fold changes. Additionally, we examined whether normalizing the whole data globally or in segments for the differential expression analysis has an effect on the performance of the normalization methods. We found that variance stabilization normalization (Vsn) reduced variation the most between technical replicates in all examined data sets. Vsn also performed consistently well in the differential expression analysis. Linear regression normalization and local regression normalization performed also systematically well. Finally, we discuss the choice of a normalization method and some qualities of a suitable normalization method in the light of the results of our evaluation.

## Introduction

The development of mass spectrometry (MS)-based proteomics has been rapid. Modern proteomics aims not only to identify the proteins but also to quantify them as accurately as possible [1]. Current MS-based proteomics workflows are able to detect thousands of proteins, their modifications and localizations in a single run [2]. Despite all the developments of MS technologies, the data from the MS analysis are still susceptible to systematic biases [3]. This bias has been defined as variation caused by nonbiological sources, which is introduced by small variations in the experimental conditions in the course of carrying out the MS analysis [4]. These variations include, for example, differences in sample preparation and handling, device calibration or changes in temperature, but the exact reason of the bias is usually unknown and cannot thus be solely accounted for by adjusting the experimental settings [3, 4]. The observed bias can be independent or dependent on the measured protein abundances [4].

The process that aims to take the bias into account is called normalization. Normalization aims to make the samples of the data more comparable and the following downstream analysis reliable [3]. Many of the normalization methods used for proteomics data have their roots in the DNA microarray technology [4], where several evaluations and reviews have already elucidated their performance [5–8]. For instance, Bolstad *et al.* [5] compared five normalization methods with DNA microarray data and concluded that most of them performed rather similarly and reduced nonbiological variability across arrays when compared with the unnormalized data. Choe *et al.* [6] also found no significant differences between the four normalization methods they examined with an RNA spike-in experiment at the probe level. In previous comparisons in proteomics, Callister *et al.* [9] used three different liquid chromatography–MS (LC-MS) data sets to evaluate four different normalization methods on peptide level and found a linear regression normalization best suited for their data sets. Kultima *et al.* [10] compared 10 different normalization methods with three different peptidomics data sets and noticed that the order of the LC-MS experiments affected the bias in the data; they suggested that their novel RegrRun normalization, which combines linear regression normalization with analysis order normalization, was the best overall method in reducing unwanted intragroup and intrasample variation.

Different tools for helping in the selection of a normalization method have also been proposed. Webb-Robertson *et al.* [11] stated that a single method cannot account for the bias in different data sets; rather it is crucial for reliable downstream analysis to select the appropriate normalization method for each data set. They introduced a tool called SPANS, which combines eight methods for peptide selection to be used in normalization with five normalization methods [11]. Chawade *et al.* [3] also introduced a tool for choosing a proper normalization method called Normalyzer. Their tool includes several popular normalization methods such as linear regression, local regression, total intensity, average intensity, median intensity, variance stabilization normalization (Vsn) and quantile normalization, together with several frequently used evaluation measures used to assess the performance of a normalization method such as the pooled coefficient of variation (PCV), the pooled median absolute deviation (PMAD) and the pooled estimate of variance (PEV) [3].

So far, comparisons of normalization methods in proteomics have typically focused on their ability to decrease intragroup variation between technical and/or biological replicates of the test data. Measures for the intragroup variation such as PEV [3, 9, 10], PCV [3], PMAD [3], the median coefficient of variation (CV) [9] and the median SD [10] have been used to rank the normalization methods compared. While reducing intragroup variation is certainly a central goal of normalization, a more thorough comparison of the normalization methods and their performance in proteomics is still lacking. Although interesting questions such as differences in the correct detection of truly differentially expressed proteins in the data normalized by different normalization methods have been investigated before [3, 12, 13], a thorough systematic analysis using multiple data sets and two-group comparisons has not been available in proteomics. Also, the effect of the normalization method on the estimation of the logarithmic fold change (logFC) or the effect of how the normalization is performed when comparing only two sample groups from a larger data set has not been systematically investigated before.

To address this need, we conducted an extensive comparison of 11 popular normalization methods or their variants. Other normalization approaches not covered in this study exist, such as the MaxLFQ integrated into the MaxQuant software [13] and the normalization integrated into the DeMix-Q software [14]. These normalizations, however, are integral parts of proteomics software workflows as opposed to the stand-alone normalization methods examined in this comparison, with the exception of Progenesis normalization. All the normalization methods examined are commonly used methods in proteomics and have different approaches and assumptions regarding the bias occurring in the data. Three spike-in label-free proteomics data sets were used for benchmarking the normalization methods. The spike-in data sets are suitable for this kind of method testing, as the differences between sample groups are known, and methods can be evaluated in their ability to find the true differences and to level out other biologically nonexisting differences. Additionally, a data set from a mouse study was also used to compare the performance of the normalization methods in a non-spike-in data set, representing a typical real research setting. Offline fractionation, which adds another layer of complexity to normalization, was not used in any of the tested data sets. In such cases, the total peptide ion signals of each fraction are spread over several runs, which should be normalized before summing up the values [12].

## Materials and methods

### Description of the data sets

#### The UPS1 data set

Benchmarking data of Pursiheimo *et al.* [15] include Universal Proteomics Standard Set (UPS1) proteins spiked into a yeast proteome digest to create concentrations of 2, 4, 10, 25 and 50 fmol/μl. Three technical replicates of each concentration were analyzed using LTQ Orbitrap Velos mass spectrometer. The spike-in data are available from the PRIDE Archive with the identifier PXD002099 (http://www.ebi.ac.uk/pride/archive/projects/PXD002099).

#### The CPTAC data set

The CPTAC (Study 6) data set [16] contains UPS1 proteins spiked into a yeast proteome digest with concentrations of 0.25, 0.74, 2.2, 6.7 and 20 fmol/μl. Three technical replicates of each concentration were analyzed using LTQ Orbitrap mass spectrometer (at test site 86). The LTQ Orbitrap@86 spike-in data are available from the CPTAC-portal (http://cptac-data-portal.georgetown.edu/cptac/dataPublic/list/LTQ-Orbitrap%4086?currentPath=%2FPhase_I_Data%2FStudy6). Sample Group E was left out from our analysis, as it had only two technical replicates because of the Progenesis software being unable to align one of the technical replicates automatically.

#### The SGSD data set

The profiling standard of Bruderer *et al.* [17] contains 12 nonhuman proteins spiked into a constant human background (HEK-293). It contains eight different sample groups with known concentrations of the spike-in proteins. Each of the samples contains three replicates, which have been analyzed both in data-dependent acquisition (DDA) and data-independent acquisition modes. We used the DDA shotgun proteomics data (referred to here as shotgun standard set, SGSD) for our comparisons. The profiling standard is available from PeptideAtlas: No. PASS00589 (username PASS00589, password WF6554orn).

#### Mouse data

The mouse data set contains liver samples of seven wild-type male mice and five transgenic male mice overexpressing cytochrome P450 aromatase [18]. The samples were analyzed with an MS/MS LTQ Orbitrap Velos Pro mass spectrometer coupled to an EASY-nLC liquid chromatography system [18]. The mouse data set is available from the ProteomeXchange with the identifier PXD002025 (http://www.ebi.ac.uk/pride/archive/projects/PXD002025). Further details of the data set are available in the original study [18].

### Common data preprocessing

The raw MS files were processed using the Progenesis QI software with the default peak-picking settings. ‘Relative quantitation using non-conflicting peptides’ setting was used, which calculates protein abundance in a run as the sum of all the unique peptide ion abundances corresponding to that protein. Peptide identifications were performed using Mascot search engine via Proteome Discoverer. For the database searches, cysteine carbamidomethylation was set as a fixed modification and methionine oxidation as a dynamic modification. Mascot score corresponding to false discovery rate of 0.01 was set as a threshold for peptide identifications.

The Progenesis software does not produce missing values *per se*, but produces some zeroes, which can be interpreted as abundance below detection capacity or protein not existing in the sample. The number of zeros in the data sets was small: 0.06–0.6% of the total of all values. As the EigenMS normalization method does not accept zero values, they were transformed into missing values (Not applicable (NA)). The same preprocessing was used with all the methods for comparability.

The exported nonnormalized data from Progenesis were transformed into log2-scale before all other normalizations except for Vsn. The Vsn normalization performs a transformation similar to the log transformation and requires the input data to be untransformed [19].

### Data analysis environment

All the data analyses were done using the R-statistical programming language version 3.2.4 [20].

### Summary of the normalization methods

#### Linear regression normalization (Rlr, RlrMA, RlrMACyc)

The linear regression normalization assumes that the bias in the data is linearly dependent on the magnitude of the measured protein intensity [9]. As the measured protein intensity increases, the bias also increases. We explored three variants of the robust linear regression called Rlr, RlrMA and RlrMA cyclic. The Rlr uses the median values over all the samples as its reference sample to which all the other samples in the data are normalized to. The RlrMA is similar, with the exception that the data are MA transformed before normalization, where A refers to the median sample and M is calculated for each sample as the difference of that sample to A. In the RlrMACyc, there is no reference, but instead, the MA transformation and the normalization of the samples are done pairwise between two samples, A being the average of the two samples and M the difference. The process is iterated through all sample pairs similar to the LinRegMA of [10]. The cycle is repeated three times, which has been observed to be enough to reach convergence between iteration cycles for the algorithm [5, 10]. All the variants of the linear regression normalizations were implemented using the robust linear regression of the R-package MASS [21]. The robust linear regression is more robust against outliers in the data than linear regression using least squares estimation. The Rlr normalization was implemented as the robust linear regression normalization of Normalyzer [3].

#### Local regression normalization (LoessF, LoessCyc)

The local regression normalization assumes a nonlinear relationship between the bias in the data and the magnitude of protein intensity [9]. We explored two common variants of local regression normalization: LoessF and LoessCyc. The data are MA transformed before normalization as with the RlrMA method. LoessF uses the mean intensities over all the samples as its reference A sample. LoessCyc is a cyclic normalization method in which two samples of the data are MA transformed and normalized at a time, and all pairs of samples are iterated through. The cycle is repeated three times similarly to the RlrMACyc method. Both of the Loess normalizations were implemented using the normalizeCyclicLoess-function from R/Bioconductor-package limma [22].

#### Variance stabilization normalization (Vsn)

The Vsn is a statistical method aiming at making the sample variances nondependent from their mean intensities and bringing the samples onto a same scale with a set of parametric transformations and maximum likelihood estimation [19]. The Vsn method was implemented with the justvsn function from the R/Bioconductor-package Vsn [19].

#### Quantile normalization (quantile)

The quantile normalization forces the distributions of the samples to be the same on the basis of the quantiles of the samples by replacing each point of a sample with the mean of the corresponding quantile [5]. The quantile normalization was performed using the normalize.quantiles function from the R/Bioconductor-package preprocessCore [23].

#### Median normalization (median)

The median normalization is based on the assumption that the samples of a data set are separated by a constant. It scales the samples so that they have the same median. The median normalization was implemented using the median intensity normalization of Normalyzer [3].

#### Progenesis normalization (Progenesis)

The Progenesis normalization is the normalization method provided by the Progenesis data analysis software. The Progenesis normalization calculates a global scaling factor between the samples by using a selected reference sample to which the other samples are normalized to. The Progenesis normalization was performed simultaneously with the preprocessing of the data.

#### EigenMS normalization (EigenMS)

The EigenMS normalization fits an analysis of variance model to the data to evaluate the treatment group effect and then uses singular value decomposition on the model residual matrix to identify and remove the bias [24]. The EigenMS aims at preserving the original differences between treatment groups while removing the bias from the data [25]. The EigenMS normalization was implemented using the R-codes of EigenMS [24] available for download in the Sourceforge-repositories (http://sourceforge.net/projects/eigenms/).

### Evaluation of the normalization methods

We evaluated the normalization methods as follows: (1) in their ability to decrease variation between technical replicates, (2) in their ability to produce data from which the truly differentially expressed proteins can be accurately found and (3) in how well the logFCs calculated from the normalized data corresponded to what was expected based on theoretical logFCs. We also evaluated whether normalizing the data globally or pairwise (i.e. based only on the sample groups under comparison) affected the performance of the methods in the differential expression analysis.

#### Intragroup variation and similarity

The effect of normalization was evaluated quantitatively using intragroup variability measures that measure the variation between technical replicates. Low intragroup variation means high similarity between technical replicates, indicating high reproducibility of the analysis. Intragroup variation was measured with PMAD, PCV and PEV. Additionally, similarity of the technical replicates in sample groups was measured with the Pearson correlation coefficient.

#### Differential expression analysis

Differential expression of proteins was examined in each two-group comparison using the reproducibility-optimized test statistic (ROTS) [26] or the *t*-test after application of the different normalization methods in each data set. The results of the differential expression analyses were evaluated with receiver operating characteristic (ROC) curve analysis, where the spike-in proteins were considered as true positives and the background proteins as true negatives. The normalization methods were ranked based on their performance in the differential expression analysis using the area under the ROC curve (AUC) as a ranking criterion. Better ranks were assigned to normalization methods with higher AUC values. In case of ties, the normalization methods received equal ranks. A mean ranking with associated standard error was calculated for each normalization method in each data set. Also, a pooled mean ranking over all the spike-in data sets was calculated for each normalization method. The Satterthwaite approximation was used to calculate the associated standard error for the pooled mean ranking. The normalization methods were ranked independently with each test statistic (ROTS, *t*-test).

#### The log fold changes of the spike-in and background proteins

The aim of normalization is to remove the unwanted (nonbiological) variation from the data. In case of the spike-in data sets used in this study, the levels of spike-in proteins should change, while the levels of the background proteins should remain unchanged. We examined the distributions of the logFCs of the spike-in and background proteins in data normalized with the different methods.

#### Evaluation of the normalization types

To explore if there is a difference in the performance of the normalization methods depending on the way in which the normalization is done, the data were normalized in two ways: globally and pairwise. In global normalization, the whole data containing all the sample groups of a data set were normalized at once. In pairwise normalization, the sample groups being compared in the differential expression analysis were first extracted from the unnormalized data and then normalized separately. Owing to the similarity of the results of the normalization types, only results of the global normalization are presented in the Results section unless where it is explicitly stated otherwise.

## Results

We examined the performance of the 11 normalization methods in three independent spike-in data sets as well as in a mouse data set from a study on changes in mouse liver lipid metabolism [18]. In the spike-in data sets, the total intensities between samples and sample groups should be almost equal. However, MS data generally show some variation in the total intensities of samples, and this was also the case in the data sets used in this study (Supplementary Figures S1–S3A). This is especially true for the UPS1 data set (Supplementary Figure S1A). After normalization, the situation is changed, and the total intensity levels of the samples are nearly equal (Supplementary Figures S1–S3). The EigenMS normalization, however, does not level the total intensities of different samples like the other normalization methods do, rather the distribution of total intensities in different samples of the EigenMS-normalized data is identical to that of the log2-transformed data.

### Effect of normalization on intragroup variation

**Figure 1:**
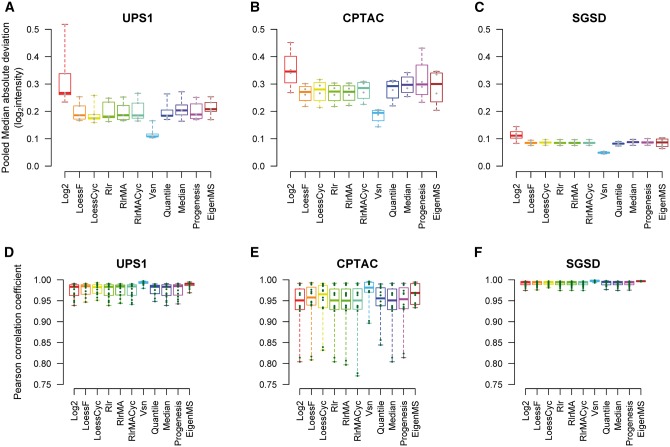
The effect of normalization method on intragroup variation between technical replicates. The PMADs of (**A**) UPS1 data, (**B**) CPTAC data and (**C**) SGSD data. The Pearson correlation coefficients of (**D**) UPS1 data, (**E**) CPTAC data and (**F**) SGSD data.

Normalization decreased intragroup variation measured as PMAD between technical replicates in all data sets when compared with the unnormalized log2-transformed data (Figure 1A–C). Vsn decreased PMAD significantly more than the other normalization methods in all data sets (Wilcoxon signed rank test *P* < 0.029 between Vsn and the other normalization methods except EigenMS in the CPTAC data set *P* = 0.057). Analogous patterns were observed also for the other intragroup variability measures (PCV and PEV) (Supplementary Figure S4A–F). Similarly, intragroup similarity between technical replicates measured with the Pearson correlation coefficient was highest in the Vsn-normalized data in all spike-in data sets (Figure 1D–F) (Wilcoxon test  <0.03 with all other methods except EigenMS in the SGSD data set *P* = 0.059 and LoessF, LoessCyc, Progenesis, quantile and EigenMS in the CPTAC-data *P* = 0.052–0.266).

### Effect of normalization on differential expression

**Figure 2:**
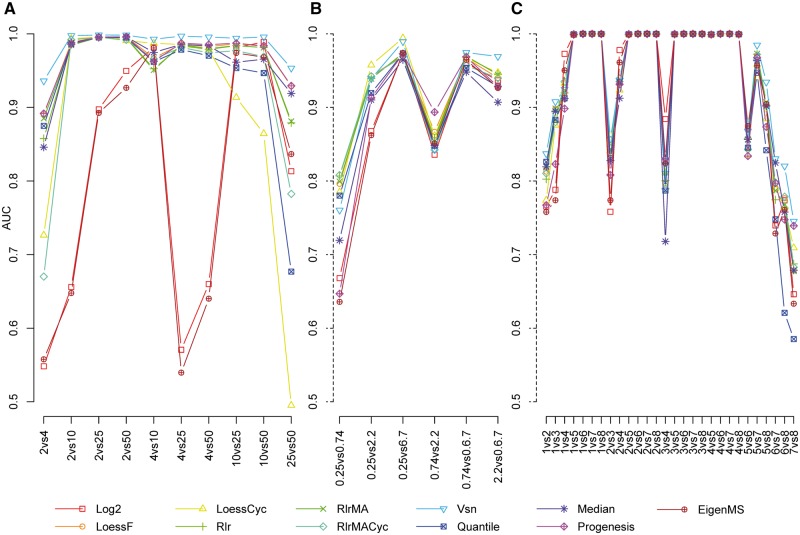
The effect of normalization method on differential expression results. The AUCs of the ROC curves of differential expression analysis in (**A**) UPS1 data, (**B**) CPTAC data and (**C**) SGSD data globally normalized with the different methods. The *x* axes denote the two-group comparisons of the sample groups.

When detecting differential expression, ROTS has been shown to perform better in proteomics data than the standard *t*-test [15], and this was the case also in the data sets used in this study (Supplementary Figure S8, Supplementary Tables S1–S2). Normalizing the data improved the AUCs of the differential expression analysis in general (Figure 2A–C, Table1). However, there was considerable variation in the performance of the different normalization methods in the different data sets tested.

**Table 1:** Rankings of the normalization methods based on AUCs of the ROC curves of the differential expression analysis using global normalization. Best mean ranking in each data set and best pooled mean ranking with each test statistic are bolded. The methods were ranked independently when using different test statistics

Normalization method	Statistical test	UPS1	CPTAC	SGSD	Pooled mean
Log2	ROTS	8 ± 1.02	8.5 ± 0.76	4.6 ± 0.78	5.9 ± 1.49
	*t*-test	7.1 ± 1.35	8 ± 1.29	4.5 ± 0.74	5.6 ± 1.87
Loess_fast	ROTS	3.5 ± 0.34	4.7 ± 1.09	4.3 ± 0.51	4.2 ± 1.25
	*t*-test	**1.9 ± 0.55**	4.7 ± 0.84	5.4 ± 0.5	4.5 ± 1.06
Loess_cyclic	ROTS	6.9 ± 1.17	**2.8 ± 0.75**	5.1 ± 0.64	5.2 ± 1.53
	*t*-test	7.9 ± 0.89	4.3 ± 0.95	7 ± 0.58	6.8 ± 1.43
Rlr_scatter	ROTS	6.4 ± 0.5	3.5 ± 0.43	4.1 ± 0.48	4.5 ± 0.82
	*t*-test	6.5 ± 0.78	4.3 ± 1.02	4.2 ± 0.46	4.7 ± 137
Rlr_ma	ROTS	6.3 ± 0.7	3.8 ± 0.48	3.9 ± 0.5	4.4 ± 0.98
	*t*-test	5.7 ± 0.68	4.3 ± 0.99	3.9 ± 0.42	4.4 ± 1.3
Rlr_ma_cyclic	ROTS	7.3 ± 0.54	6.5 ± 1.57	4.3 ± 0.58	5.3 ± 1.75
	*t*-test	6.3 ± 0.67	4.2 ± 1.45	4.6 ± 0.5	4.9 ± 1.68
Vsn	ROTS	**1 ± 0**	4.3 ± 1.41	**2.7 ± 0.46**	**2.5 ± 1.48**
	*t*-test	3.9 ± 0.31	**3.8 ± 0.87**	**3.5 ± 0.34**	**3.6 ± 0.98**
Quantile	ROTS	8.2 ± 0.61	7.7 ± 0.56	7 ± 0.74	7.4 ± 1.11
	*t*-test	8.8 ± 0.39	8.3 ± 0.67	9.6 ± 0.46	9.2 ± 0.99
Median	ROTS	5.9 ± 0.75	9.5 ± 0.72	5 ± 0.68	5.8 ± 1.24
	*t*-test	6.2 ± 0.98	10 ± 0.54	5.3 ± 0.65	6.2 ± 1.16
Progenesis	ROTS	3.3 ± 0.67	6.7 ± 1.41	6.1 ± 0.75	5.5 ± 1.73
	*t*-test	3 ± 0.54	5.3 ± 1.17	7.1 ± 0.59	5.9 ± 1.41
EigenMS	ROTS	9.2 ± 0.76	8 ± 1.32	5.5 ± 0.85	6.7 ± 1.74
	*t*-test	8.6 ± 0.92	8.5 ± 1.18	5.3 ± 0.8	6.5 ± 1.51

The benefits of normalization were most prominent in the UPS1 data set (Figure 2A, Table 1), in which all the other normalization methods were ranked higher than the simple log2 transformation except for the EigenMS and the Quantile normalization. The Vsn-normalized data had the highest AUC in every two-group comparison in the UPS1 data set when using ROTS (Delong’s test *P* < 0.04 with all the other methods).

In the CPTAC and SGSD data sets, the differences between the normalization methods were smaller on average, but some differences were found. In the CPTAC data set, all the normalization methods, except for the median normalization, ranked on average higher than the log2 transformation when the differential expression was analyzed with ROTS (Figure 2B, Table 1). In most of the two-group comparisons in the CPTAC data, no significant differences in the AUCs produced by the best ranking normalization method and the other methods were observed (Delong’s test *P* > 0.05), with few exceptions. In the 0.74 versus 2.2 fmol comparison, the Progenesis normalization ranked first and gave a significantly higher AUC than 8 of 10 methods (Delong’s test *P* < 0.049). In the 2.2 versus 6.7 fmol comparison, the Vsn normalization ranked first and gave a significantly higher AUC than 6 of 10 methods (Delong’s test *P* < 0.028). In the 0.25 versus 0.74 comparison, the RlrMACyc normalization method ranked best and gave an AUC significantly higher than half of the other methods (Delong’s test *P* < 0.044 for 5 of 10 methods).

In the SGSD data set, differences between the different normalization methods and the log2 transformation were generally small. Only five normalization methods, the Vsn, RlrMA, Rlr, RlrMACyc and LoessF, ranked on average higher than the log2 transformation in the SGSD data set (Table 1). In most of the two-group comparisons, there was no significant difference between the AUC of the best ranking method and the AUCs of the other methods (Delong’s test *P* > 0.05), with few exceptions. In the 5 versus 7, 5 versus 8, 6 versus 7, 6 versus 8 and 7 versus 8 comparisons, the Vsn normalization consistently ranked first and gave a higher AUC than most of the other methods tested (Delong’s test *P* < 0.046 for 6–8 of 10 methods; Figure 2C).

While no single method gave the highest AUC in every two-group comparison, the Vsn normalization performed consistently well, giving high AUCs throughout all data sets. This resulted in the highest pooled mean rank across all data sets and high mean ranks regardless of the test statistic used (Table 1). The linear regression methods relying on an artificial reference (RlrMA and Rlr) and the local regression method using an artificial reference (LoessF) also performed systematically well throughout all the comparisons in all data sets (Figure 2, Table 1). Some of the visuals are overlapping in Figure 2. In particular, LoessF is covered largely by the lines of the other normalization methods: Progenesis normalization in Figure 2A and other methods in Figures 2B and C.

### Effect of normalization type

In general, whether the data were normalized globally or pairwise between the two groups compared did not have a major effect on the AUCs of the differential expression analysis (Figure 2A–C versus Supplementary Figure S5A–C). The only exceptions were the cyclic normalization methods, LoessCyc and RlrMACyc, which benefitted from normalizing the data pairwise in the UPS1 data set (Figure 2A versus Supplementary Figure S5A). This could also be seen in the MA plots of the UPS1 data, in which the data were centered well in the line M = 0 in the pairwise normalized data of the cyclic methods, but not in the globally normalized data of the same methods (Supplementary File S1).

### Effect of normalization on logFC

**Figure 3:**
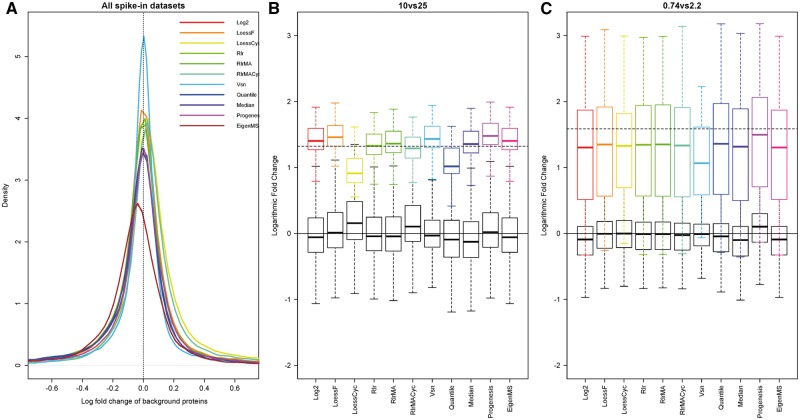
The logFC of the background proteins and representative examples of the logFC of the spike-in proteins. (**A**) The density distributions of the logFC of the background proteins over all two-group comparisons in all data sets. The vertical dashed line corresponds to logFC of zero. The logFC of the spike-in proteins (upper boxes) and the background proteins (lower boxes) in the (**B**) 10 versus 25 fmol comparison of the UPS1 data and (**C**) the 0.74 versus 2.2 fmol comparison of the CPTAC data. The horizontal solid black lines correspond to logFC of zero, while the horizontal dashed lines correspond to the theoretical expected logFC of the spike-in proteins.

When looking at the distribution of the logFC of the background proteins in all data sets, we can see that it is centered around zero for all the other normalization methods except for the EigenMS normalization (Figure 3A), for which the distribution was identical to that of the log2 transformation. The distribution of the logFCs in the Vsn-normalized data was more concentrated around zero than in data sets normalized with the other methods, which can be seen as a narrower and higher density distribution for the Vsn-normalized data.

Based on the known concentrations of the spike-in proteins, the logFCs of the spike-in proteins were typically underestimated both in the normalized data as well as in the log2-transformed data (Figure 3B and C, Supplementary File S2). The EigenMS-normalized data gave similar estimates as the log2-transformed data; the Vsn normalization gave generally more conservative estimates than the other normalization methods. All the other normalization methods gave consistently similar estimates for the logFC of the spike-in proteins. In the UPS1 data, the logFC of the spike-in proteins of the normalized data was closer to the theoretical known logFC in general than in the log2-transformed data (Supplementary File S2).

### Visual quality inspection

**Figure 4:**
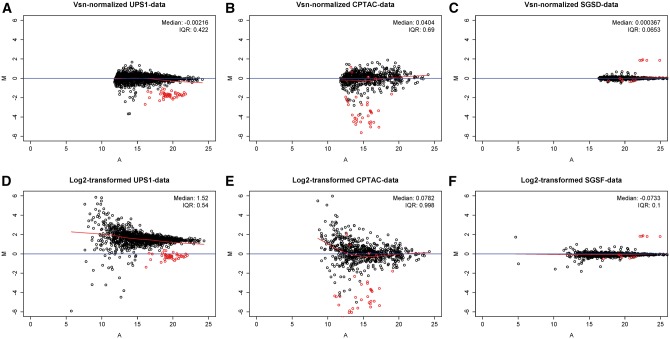
Representative MA plots of the two-group comparisons after normalization with the most successful normalization method and log2 transformation in each data set. MA plots of the (**A**) 2 versus 10 fmol comparison of the UPS1 data, (**B**) 0.25 versus 2.2 fmol comparison of the CPTAC data and (**C**) sample 1 versus sample 4 comparison of the SGSD data normalized with the Vsn normalization. MA plots of the (**D**) 2 versus 10 fmol comparison of the UPS1 data, (**E**) 0.25 versus 2.2 fmol comparison of the CPTAC data and (**F**) sample 1 versus sample 4 comparison of the SGSD data after the log2 transformation. The lighter nonblack points in the plots correspond to the spike-in proteins and the black points to the background proteins. The curve corresponds to a loess smoothing function.

The MA plot is a common tool for exploring the bias in the data of two samples [5, 9]. Normalization aims to remove the bias from the data and center the data scatter of the sample pair examined around the *x* axis (M = 0) in the MA plot. In this study, MA plots were drawn and observed with each normalization method in each two-group comparison of each data set. Based on visual inspection of these plots, the Vsn normalization seems to concentrate the data more tightly both around the *x* axis and to a narrower scale of transformed intensities than the logarithm transformation and the other normalization methods in general (Figure 4, Supplementary File S1). In the CPTAC and the SGSD data sets, the data in the two-group comparisons were well centered already after the logarithm transformation. In the UPS1 data, the data after the cyclic normalizations (RlrMACyc and LoessCyc) were much more centered after pairwise normalization than after global normalization (Supplementary File S1). In many two-group comparisons, the quantile normalization seemed to introduce extra patterns into the data on high intensities not seen in the unnormalized log2-transformed data (Supplementary File S1).

### Testing on mouse data

In addition to the three spike-in data sets, we also compared the performance of the normalization methods in a mouse study data set, which represents a typical real study setting [18]. When looking at the levels of total intensities of the samples in the log2-transformed mouse data, we can see that they are unequal (Supplementary Figure S6A). When applying normalization, most of the methods equalize the levels of total intensities of different samples, except for the EigenMS (Supplementary Figure S6B–K).

**Figure 5:**
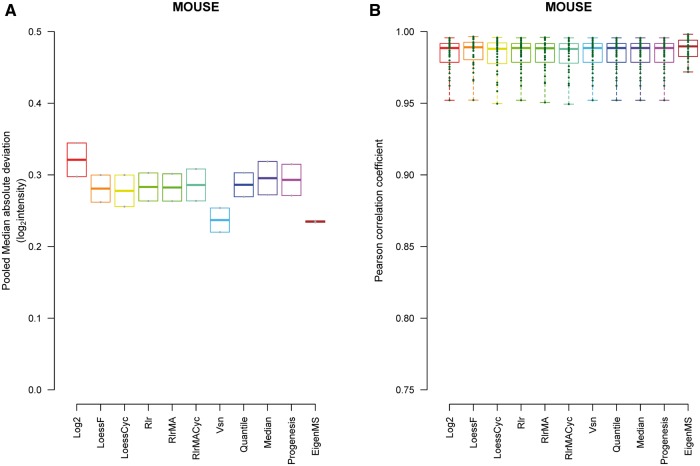
Intragroup variation between biological replicates in the mouse data normalized with the different methods. (**A**) The PMADs and (**B**) the Pearson correlation coefficients.

In the mouse data set, we investigated biological replicates of the same treatment group instead of technical replicates. Similar patterns for intragroup variation for data normalized with the different methods were observed as with the spike-in data sets (Figure 5). All normalization methods decreased intragroup variation when measured with the PMAD compared with the unnormalized data. PMAD was smallest in the Vsn- and EigenMS-normalized data, but the differences to the other methods were not significant (Wilcoxon signed rank test  >0.33; Figure 5A). Similar patterns were observed with the other intragroup measures PCV and PEV (Supplementary Figure S7). Intragroup similarity measured with the Pearson correlation coefficient was highest among the EigenMS-normalized data, but the differences to the other methods were small (Wilcoxon *P* > 0.18; Figure 5B). The mouse data did not contain any spike-in proteins and thus we did not have prior knowledge about expected protein changes. Therefore, differential expression analysis was not directly applicable to assess the performance of the normalization methods. The same was true for the logFC.

## Discussion

In the spike-in data sets examined in this study, the Vsn normalization consistently reduced intragroup variation the most, increased intragroup similarity the most and gave consistently high AUCs in the differential expression analysis, resulting in the highest pooled mean ranking among the normalization methods tested. The EigenMS normalization also consistently reduced intragroup variation more than the other methods examined, but it did not perform well in the differential expression analysis. Also, other normalization methods decreased intragroup variation when compared with the unnormalized log2-transformed data, but no major differences between them were observed. In previous comparisons of normalization methods in proteomics/peptidomics focusing on intragroup variation measures, the Vsn normalization has been ranked average [10] or as among the most suitable methods [3]. Previous studies have suggested the linear regression normalization or its variants or local regression normalization to reduce intragroup variation the most [3, 9, 10]. We observed the linear regression normalization variants and the local regression normalization variants performing on par with the other normalization methods in reducing intragroup variation, with no major differences. However, even though we did not observe the linear and local regression to reduce intragroup variation more than the other normalization methods, we noticed that the local regression method using a mean reference sample, LoessF, consistently produced high AUCs in the differential expression analysis. The same was true for the linear regression methods using a median reference sample, Rlr and RlrMA. The local regression normalization fared better in the UPS1 data set, while the linear regression normalization performed better in the CPTAC and SGSD data sets, perhaps indicating a different kind of bias in the data sets. Typically, the variants using a reference sample performed better than their cyclic counterparts, with the exception of the cyclic loess normalization LoessCyc in the CPTAC data.

It became clear that the spike-in data sets in this analysis differed from each other. The sample groups of the UPS1 data set had much larger variation in the total intensities than the other two data sets, especially the SGSD data set, which had many sample groups with roughly similar levels of total intensities. This could be because of a number of reasons, such as different instrumentation or protocols/methods used, but is interesting from the point of normalization. The total intensities between the samples may vary from data to data also in the case of real experimental study settings, and we would like to find a normalization method that can perform as consistently as possible no matter the characteristics of the data. Notably, normalization clearly improved the AUCs also in the CPTAC data set when compared with the unnormalized log2-transformed data (Table 1), regardless of the fact that it had rather equal total intensity levels before normalization. This emphasizes the importance of a consistent normalization method; even if we have a high-quality data set with rather equal unnormalized sample levels, we cannot necessarily deduce whether a simple logarithmic transformation would suffice in delivering the truly differentially expressed proteins reliably. Also, the nature of the bias might be different in different data sets. Therefore, the used normalization should not make too rigid assumptions about the nature of the bias, unless we know or can estimate the bias and purposefully want to use a method targeting specifically that kind of bias. The Vsn, quantile and the EigenMS normalizations do not make strict assumptions about the nature of the bias and are general methods in that sense.

The median and quantile normalizations were on par with most of the normalization methods in reducing intragroup variation, but they did not rank well in terms of differential expression analysis. It is notable, however, that even though not having a high ranking, both methods performed consistently in the differential expression analysis by not producing low AUCs in any of the two-group comparisons like the log2 transformation did in the UPS1 data set (Figure 2A). More worrying is the tendency of the quantile normalization to introduce extra patterns into the data on high intensities seen on many two-group comparisons (Supplementary File S1). The Progenesis normalization had the second highest ranking in the differential expression analysis in the UPS1 data, but ranked worse in the two other data sets examined (Table 1). The EigenMS behaved differently from the other normalization methods examined in this study. While it was effective in reducing intragroup variation, it did not perform so well in the differential expression analysis. Instead, it performed similarly as the simple log2 transformation.

An arbitrary but commonly used cutoff value to determine differentially expressed genes and proteins is a logFC of one [27–29], which corresponds to a 2-fold change in expression. As we noticed from the logFC plots of the data normalized with the different methods (Figure 3B and C, Supplementary File S2), the estimates for the known differentially expressed proteins frequently remained under this limit even if the differentially expressed proteins were detected with great accuracy. This was especially true for the Vsn-normalized data, which gave conservative estimates for the logFC of the spike-in proteins, but from which the spike-in proteins were detected with great accuracy. This warrants caution for the use of any such generic cutoff values for filtering the differentially expressed proteins based on their logFC.

Although Vsn performed generally well in our comparisons, the fact that it consistently underestimated the logFCs of the spike-in proteins can be seen as a potential drawback of the method if the researcher would be interested particularly in examining the logFCs of proteins. For this particular task, some of the other well-performing normalization methods (LoessF, Rlr, RlrMA) would be perhaps more suitable. Also, all of the normalization methods studied here, excluding EigenMS, assume that only a small portion of the proteins are differentially expressed between samples and force the total intensity levels of the samples to be on the same level (Supplementary Figure S1). This might be problematic if in fact a large number of proteins are differentially expressed between samples. In such cases, methods like the EigenMS might be more suitable for normalizing the data. We encourage the researcher to reflect on what is known beforehand about the task at hand and select the appropriate normalization method accordingly.

All of the normalizations in this study were performed on protein-level data. Normalization can be performed also at the peptide level. The next step would be to perform a similar exhaustive comparison of the normalization methods on peptide level and explore if the same methods fare well with peptide data. Also, the choice of peptides to be used for the normalization has been demonstrated to have an effect [11], and exploring this idea in conjunction with the normalizations used in this study would be an interesting further topic.

Based on the comparisons made in this study, normalization decreased intragroup variation in general and resulted in better AUCs in the differential expression analysis than the simple log2 transformation in case of most of the normalization methods examined. The Vsn normalization performed consistently well in reducing intragroup variation and in the differential expression analysis in all tested data sets. The local regression and linear regression normalizations using a reference also reduced intragroup variation compared with the unnormalized data and consistently delivered good AUCs in the differential expression analysis.

**Key Points**

- Data generated by the MS analysis are prone to biases, which can be accounted for with normalization resulting in more reliable downstream analysis.
- In total, 11 normalization methods were systematically evaluated in this study using three spike-in and a mouse label-free proteomics data sets.
- Vsn reduced variation the most between the technical replicates in all studied data sets and consistently performed well in the differential expression analysis. The local regression normalization using an artificial reference sample (LoessF) and linear regression normalization using artificial reference samples (Rlr and RlrMA) also performed systematically well in the differential expression analysis.
- The nature and extent of the bias in the data are not generally known beforehand; the application of a consistent normalization method is crucial for reliable results.

## Supplementary Data

Supplementary data are available online at http://bib.oxfordjournals.org/.

## Funding

This study was supported by the Sigrid Juselius Foundation, JDRF [grant number 2-2013-32] and the European Research Council (ERC) Starting Grant no. 677943.

## Supplementary Material

Supplementary Figures

Supplementary File1

Supplementary File2

Supplementary Table1

Supplementary Table2

## Notes

**Tommi Välikangas** is a Research Scientist in the Computational Biomedicine Group at the Turku Centre for Biotechnology Finland. He is interested in computational biology and bioinformatics.

## Notes

**Tomi Suomi** is a Research Scientist in the Computational Biomedicine research group at the Turku Centre for Biotechnology Finland. His research interests include scientific computing and bioinformatics.

## Notes

**Laura L. Elo** is Adjunct Professor in Biomathematics, Research Director in Bioinformatics and Group Leader in Computational Biomedicine at Turku Centre for Biotechnology, University of Turku, Finland. Her main research interests include computational biomedicine and bioinformatics.
