# Supplementary material for: A systematic evaluation of normalization methods in quantitative label-free proteomics
Source: Brief Bioinform. 2016 Oct 2;19(1):1–11. doi: 10.1093/bib/bbw095 (PMC5862339; doi:10.1093/bib/bbw095)
Supplement: Supplementary Figures [file bbw095_supplementary_figures.docx]

Supplementary Figure 1. **The total intensities of samples of the unnormalized UPS1-data and data normalized with the different methods.** The total intensities of samples of the (A) log-transformed, (B) LoessF-normalized, (C) LoessCyc-normalized, (D) Rlr-normalized, (E) RlrMA-normalized, (F) RlrMACyc-normalized, (G) Vsn-normalized, (H) Quantile-normalized, (I) Median-normalized, (J) Progenesis-Normalized, and (K) EigenMS-normalized UPS1-data.

Supplementary Figure 2. **The total intensities of samples of the unnormalized CPTAC-data and data normalized with the different methods.** The total intensities of samples of the (A) log-transformed, (B) LoessF-normalized, (C) LoessCyc-normalized, (D) Rlr-normalized, (E) RlrMA-normalized, (F) RlrMACyc-normalized, (G) Vsn-normalized, (H) Quantile-normalized, (I) Median-normalized, (J) Progenesis-Normalized, and (K) EigenMS-normalized CPTAC-data.

Supplementary Figure 3. **The total intensities of samples of the unnormalized SGSD-data and data normalized with the different methods.** The total intensities of samples of the (A) log-transformed, (B) LoessF-normalized, (C) LoessCyc-normalized, (D) Rlr-normalized, (E) RlrMA-normalized, (F) RlrMACyc-normalized, (G) Vsn-normalized, (H) Quantile-normalized, (I) Median-normalized, (J) Progenesis-Normalized, and (K) EigenMS-normalized SGSD-data.


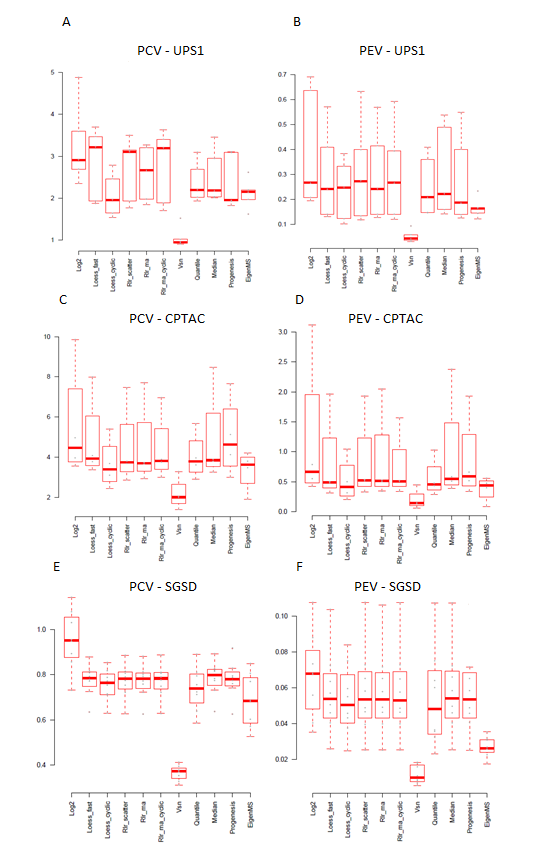


Supplementary Figure 4. **The Pooled Coefficients of Variation (PCV) and the Pooled Estimates of Variance (PEV) of intragroup (between technical replicates) variation in data normalized with the different methods.** (A) The PCV of UPS1-data, (B) PEV of UPS1-data, (C) PCV of CPTAC-data (D) PEV of CPTAC-data, (E) PCV of SGSD-data, and (F) PEV of SGSD-data.

Supplementary Figure 5. **The areas under the ROC-curves (AUC) of differential expression analysis using pairwise normalization**. AUCs in (A) UPS1-data, (B) CPTAC-data and (C) SGSD-data pairwise normalized with the different methods. The x-axes denote the two-group comparisons of the sample groups.

Supplementary Figure 6. **The total intensities of samples of the unnormalized Mouse-data and data normalized with the different methods.** The total intensities of samples of the (A) log-transformed, (B) LoessF-normalized, (C) LoessCyc-normalized, (D) Rlr-normalized, (E) RlrMA-normalized, (F) RlrMACyc-normalized, (G) Vsn-normalized, (H) Quantile-normalized, (I) Median-normalized, (J) Progenesis-Normalized, and (K) EigenMS-normalized Mouse-data.


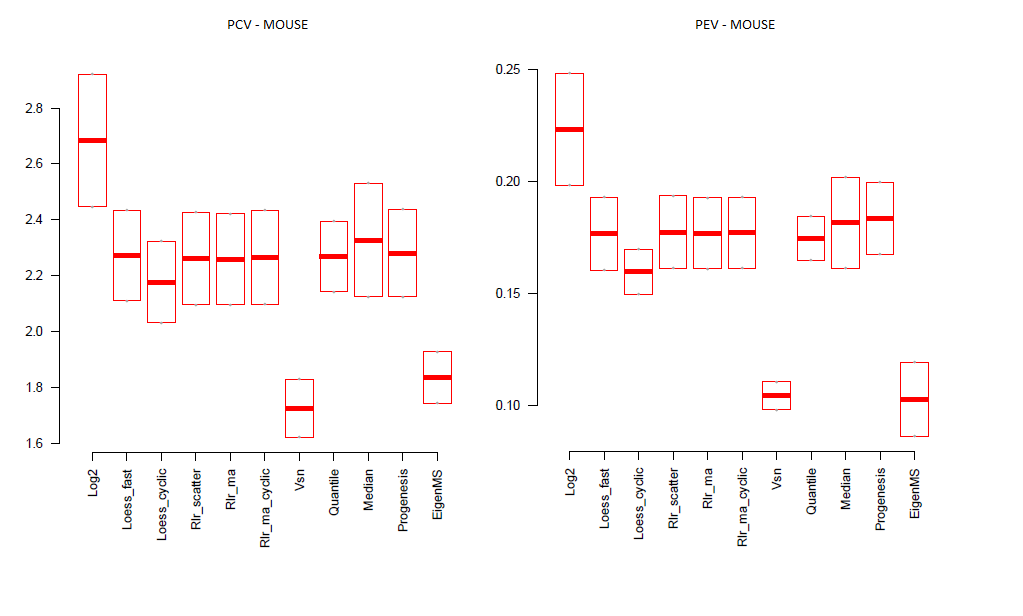


Supplementary Figure 7. **The Pooled Coefficients of Variation (PCV) and the Pooled Estimates of Variance (PEV) of intragroup (between technical replicates) variation in mouse data normalized with the different methods.** (A) The PCV, and (B) PEV of the mouse-data

A

B

Supplementary Figure 8. **Median areas under the ROC-curves (AUC) over all the normalization methods over all the pairwise comparisons by ROTS and t-test in each dataset** in (A) globally normalized data, and (B) pairwise normalized data. The whiskers represent the median absolute deviations (MAD).
