# Supplementary material for: A systematic evaluation of normalization methods in quantitative label-free proteomics
Source: Brief Bioinform. 2016 Oct 2;19(1):1–11. doi: 10.1093/bib/bbw095 (PMC5862339; doi:10.1093/bib/bbw095)
Supplement: Supplementary File1 [file bbw095_supplementary_file1.docx]

Supplementary File 1. **MA-plots of each two-group comparison after normalization by the different methods and log2-transformation in each dataset**. The x-axis represents the average protein abundance and the y-axis the ratio of the proteins as in Figure 4. The red line corresponds to a loess smoothing function. The normalization type (global or pairwise normalization) is indicated in the lower right corner of each figure.
