# Supplementary material for: A systematic evaluation of normalization methods in quantitative label-free proteomics
Source: Brief Bioinform. 2016 Oct 2;19(1):1–11. doi: 10.1093/bib/bbw095 (PMC5862339; doi:10.1093/bib/bbw095)
Supplement: Supplementary File2 [file bbw095_supplementary_file2.docx]

Supplementary File 2. **The logarithmic fold change (LogFC) of the spike-in (coloured boxes) and background proteins (black boxes).** The horizontal solid black lines correspond to LogFC of zero while the horizontal dashed lines correspond to the theoretical expected LogFC of the spike-in proteins.
