# Supplementary material for: A systematic evaluation of normalization methods in quantitative label-free proteomics
Source: Brief Bioinform. 2016 Oct 2;19(1):1–11. doi: 10.1093/bib/bbw095 (PMC5862339; doi:10.1093/bib/bbw095)
Supplement: Supplementary Table1 [file bbw095_supplementary_table1.docx]

| Supplementary Table 1. **Areas under the ROC-curves (AUC) of the differential expression analysis using global normalization and different test statistics.** Differential expression analyzed with ROTS and the common t-test. | | | | | | | | | | | |
| --- | --- | --- | --- | --- | --- | --- | --- | --- | --- | --- | --- |
|  |  |  |  |  |  |  |  |  |  |  |  |
|  | UPS1 GLOBAL ROTS | |  |  |  |  |  |  |  |  |  |
| Comparison pair | Log2 | LoessF | LoessCyc | Rlr | RlrMA | RlrMACyc | Vsn | Quantile | Median | Progenesis | EigenMS |
| 2 vs. 4 | 0,548168 | 0,890336 | 0,726419 | 0,857812 | 0,885059 | 0,670128 | 0,935984 | 0,874962 | 0,845922 | 0,891672 | 0,557719 |
| 2 vs. 10 | 0,655862 | 0,988353 | 0,993343 | 0,98525 | 0,984589 | 0,991879 | 0,997474 | 0,985979 | 0,987946 | 0,9861151 | 0,647745 |
| 2 vs. 25 | 0,897395 | 0,995934 | 0,995591 | 0,995358 | 0,995223 | 0,995155 | 0,998374 | 0,995121 | 0,994952 | 0,995477 | 0,89258 |
| 2 vs. 50 | 0,949589 | 0,995884 | 0,990764 | 0,994677 | 0,994268 | 0,991853 | 0,998265 | 0,994932 | 0,995867 | 0,9960882 | 0,926648 |
| 4 vs. 10 | 0,981603 | 0,967035 | 0,98714 | 0,952653 | 0,950806 | 0,965815 | 0,992716 | 0,961936 | 0,974709 | 0,9626304 | 0,980832 |
| 4 vs. 25 | 0,570693 | 0,985883 | 0,985519 | 0,983547 | 0,985325 | 0,981499 | 0,996852 | 0,978503 | 0,985612 | 0,9873051 | 0,539607 |
| 4 vs. 50 | 0,659931 | 0,982649 | 0,978253 | 0,978927 | 0,979896 | 0,974152 | 0,995616 | 0,97026 | 0,983822 | 0,9854191 | 0,640153 |
| 10 vs. 25 | 0,981296 | 0,986476 | 0,913465 | 0,983886 | 0,984072 | 0,977149 | 0,993991 | 0,953452 | 0,961458 | 0,9872374 | 0,97402 |
| 10 vs. 50 | 0,988979 | 0,983094 | 0,864429 | 0,981343 | 0,981292 | 0,968978 | 0,995714 | 0,946715 | 0,965525 | 0,9837237 | 0,968196 |
| 25 vs. 50 | 0,813275 | 0,928745 | 0,495147 | 0,878969 | 0,881156 | 0,782504 | 0,953378 | 0,676884 | 0,918912 | 0,929338 | 0,836624 |
|  |  |  |  |  |  |  |  |  |  |  |  |
| Comparison pair | CPTAC GLOBAL ROTS | | |  |  |  |  |  |  |  |  |
|  | Log2 | LoessF | LoessCyc | Rlr | RlrMA | RlrMACyc | Vsn | Quantile | Median | Progenesis | EigenMS |
| 0.25 vs. 0.74 | 0,66802 | 0,796243 | 0,784969 | 0,806909 | 0,799308 | 0,807983 | 0,760387 | 0,780126 | 0,719476 | 0,6468481 | 0,635785 |
| 0.25 vs. 2.2 | 0,867926 | 0,942691 | 0,957633 | 0,939343 | 0,938945 | 0,942372 | 0,938905 | 0,919576 | 0,910171 | 0,9130002 | 0,862307 |
| 0.25 vs 6.7 | 0,97314 | 0,970506 | 0,994337 | 0,97338 | 0,973819 | 0,969149 | 0,989464 | 0,97306 | 0,964559 | 0,9726612 | 0,974101 |
| 0.74 vs 2.2 | 0,835685 | 0,86677 | 0,861614 | 0,85856 | 0,854137 | 0,84238 | 0,842938 | 0,84788 | 0,848278 | 0,8935916 | 0,851148 |
| 0.74 vs 6.7 | 0,960192 | 0,966707 | 0,967043 | 0,969185 | 0,969704 | 0,957074 | 0,97458 | 0,953797 | 0,948042 | 0,9685052 | 0,964708 |
| 2.2 vs 6.7 | 0,936679 | 0,940162 | 0,9472 | 0,945365 | 0,944565 | 0,939761 | 0,96922 | 0,928474 | 0,90702 | 0,9283942 | 0,926433 |
|  |  |  |  |  |  |  |  |  |  |  |  |
|  | SGSD GLOBAL ROTS | |  |  |  |  |  |  |  |  |  |
| Comparison pair | Log2 | LoessF | LoessCyc | Rlr | RlrMA | RlrMACyc | Vsn | Quantile | Median | Progenesis | EigenMS |
| 1 vs. 2 | 0,765271 | 0,812842 | 0,773851 | 0,802547 | 0,820697 | 0,810256 | 0,837041 | 0,826015 | 0,819331 | 0,7672229 | 0,757909 |
| 1 vs .3 | 0,788105 | 0,897834 | 0,875953 | 0,893882 | 0,898566 | 0,880123 | 0,907787 | 0,883148 | 0,894809 | 0,8230386 | 0,773823 |
| 1 vs. 4 | 0,972824 | 0,920326 | 0,932796 | 0,91974 | 0,920228 | 0,935158 | 0,924522 | 0,912324 | 0,912227 | 0,8983216 | 0,9504 |
| 1 vs. 5 | 0,998927 | 0,999463 | 0,999511 | 0,999561 | 0,999561 | 0,999122 | 0,999415 | 0,999561 | 0,998878 | 0,9993657 | 0,998438 |
| 1 vs. 6 | 1 | 1 | 1 | 0,999951 | 0,999951 | 0,999854 | 0,999902 | 1 | 0,999951 | 1 | 1 |
| 1 vs. 7 | 1 | 1 | 1 | 1 | 1 | 1 | 1 | 1 | 1 | 1 | 1 |
| 1 vs. 8 | 0,999707 | 0,999902 | 0,999902 | 0,999902 | 0,999902 | 0,999902 | 0,999854 | 0,999756 | 0,999902 | 0,9998535 | 0,999902 |
| 2 vs. 3 | 0,758246 | 0,838017 | 0,855523 | 0,838456 | 0,843677 | 0,844945 | 0,857289 | 0,830699 | 0,827674 | 0,8082065 | 0,773921 |
| 2 vs. 4 | 0,977947 | 0,933011 | 0,92346 | 0,934914 | 0,933206 | 0,939305 | 0,936719 | 0,934182 | 0,912471 | 0,93145 | 0,96114 |
| 2 vs. 5 | 0,999463 | 0,999561 | 0,999609 | 0,999561 | 0,999561 | 0,999561 | 0,999561 | 0,999512 | 0,999658 | 0,9994145 | 0,998682 |
| 2 vs. 6 | 1 | 1 | 1 | 1 | 1 | 1 | 1 | 1 | 1 | 1 | 1 |
| 2 vs. 7 | 1 | 0,999902 | 0,999951 | 0,999951 | 0,999951 | 0,999951 | 1 | 1 | 1 | 0,9999024 | 0,999902 |
| 2 vs. 8 | 0,999902 | 0,999805 | 0,999804 | 0,999805 | 0,999805 | 0,999854 | 0,999854 | 0,999756 | 0,999805 | 0,9996095 | 0,999805 |
| 3 vs. 4 | 0,884319 | 0,796399 | 0,810899 | 0,800351 | 0,798009 | 0,789422 | 0,810353 | 0,78708 | 0,717896 | 0,830162 | 0,82396 |
| 3 vs. 5 | 0,999512 | 0,99961 | 0,99956 | 0,999707 | 0,999707 | 0,999707 | 0,999658 | 0,999415 | 0,999561 | 0,9998048 | 0,999121 |
| 3 vs. 6 | 1 | 1 | 1 | 1 | 1 | 1 | 1 | 0,999902 | 0,999902 | 0,9998536 | 1 |
| 3 vs. 7 | 1 | 1 | 1 | 1 | 1 | 1 | 1 | 0,999902 | 1 | 1 | 1 |
| 3 vs. 8 | 0,999951 | 0,999756 | 0,999804 | 0,999707 | 0,999707 | 0,999707 | 0,999756 | 0,999756 | 0,999805 | 0,9997559 | 0,999805 |
| 4 vs. 5 | 0,999171 | 0,998975 | 0,998778 | 0,999024 | 0,999024 | 0,999024 | 0,999463 | 0,999024 | 0,999024 | 0,9989754 | 0,999219 |
| 4 vs. 6 | 1 | 1 | 1 | 1 | 1 | 1 | 1 | 1 | 1 | 1 | 1 |
| 4 vs. 7 | 1 | 1 | 1 | 1 | 1 | 1 | 1 | 0,999854 | 1 | 1 | 0,999951 |
| 4 vs. 8 | 0,999317 | 0,999365 | 0,999413 | 0,999365 | 0,999365 | 0,999365 | 0,999414 | 0,999268 | 0,999561 | 0,9994142 | 0,999658 |
| 5 vs. 6 | 0,871438 | 0,845921 | 0,835239 | 0,858167 | 0,856021 | 0,842994 | 0,86773 | 0,845238 | 0,856557 | 0,8339188 | 0,87478 |
| 5 vs. 7 | 0,969262 | 0,96853 | 0,958358 | 0,972336 | 0,973019 | 0,964627 | 0,984582 | 0,94716 | 0,963603 | 0,9676034 | 0,956893 |
| 5 vs. 8 | 0,903827 | 0,90495 | 0,884604 | 0,902949 | 0,907196 | 0,9029 | 0,934339 | 0,841779 | 0,901875 | 0,8735599 | 0,904511 |
| 6 vs. 7 | 0,739657 | 0,790837 | 0,789492 | 0,774493 | 0,786934 | 0,798107 | 0,830211 | 0,747902 | 0,824649 | 0,7970336 | 0,728715 |
| 6 vs. 8 | 0,773726 | 0,776606 | 0,76437 | 0,763327 | 0,768209 | 0,778705 | 0,820348 | 0,620826 | 0,757909 | 0,7476079 | 0,760935 |
| 7 vs. 8 | 0,646163 | 0,678481 | 0,709238 | 0,687366 | 0,677456 | 0,682874 | 0,745118 | 0,585286 | 0,678871 | 0,7391135 | 0,633275 |

|  | UPS1 GLOBAL TTEST | |  |  |  |  |  |  |  |  |  |
| --- | --- | --- | --- | --- | --- | --- | --- | --- | --- | --- | --- |
| Comparison pair | Log2 | LoessF | LoessCyc | Rlr | RlrMA | RlrMACyc | Vsn | Quantile | Median | Progenesis | EigenMS |
| 2 vs. 4 | 0,882461 | 0,699386 | 0,583233 | 0,674161 | 0,699697 | 0,578399 | 0,678299 | 0,677849 | 0,538088 | 0,7266285 | 0,603501 |
| 2 vs. 10 | 0,53062 | 0,986115 | 0,966491 | 0,976634 | 0,975632 | 0,985061 | 0,985821 | 0,969926 | 0,970261 | 0,9844916 | 0,595848 |
| 2 vs. 25 | 0,913512 | 0,99551 | 0,987488 | 0,99266 | 0,992781 | 0,992229 | 0,994664 | 0,979138 | 0,994007 | 0,9950435 | 0,887701 |
| 2 vs. 50 | 0,812195 | 0,994828 | 0,991026 | 0,990867 | 0,98976 | 0,993738 | 0,992596 | 0,983377 | 0,994586 | 0,9939284 | 0,669365 |
| 4 vs. 10 | 0,969955 | 0,884351 | 0,915608 | 0,813023 | 0,830628 | 0,892347 | 0,896534 | 0,850961 | 0,846242 | 0,8819321 | 0,979944 |
| 4 vs. 25 | 0,673257 | 0,917476 | 0,903741 | 0,844718 | 0,870134 | 0,885527 | 0,911233 | 0,834439 | 0,912414 | 0,9174762 | 0,796448 |
| 4 vs. 50 | 0,64219 | 0,910487 | 0,857929 | 0,843469 | 0,868654 | 0,857476 | 0,902155 | 0,812471 | 0,904075 | 0,9066127 | 0,755551 |
| 10 vs. 25 | 0,983071 | 0,980658 | 0,881227 | 0,975471 | 0,976002 | 0,975557 | 0,980024 | 0,935006 | 0,946697 | 0,9804009 | 0,972438 |
| 10 vs. 50 | 0,967065 | 0,985941 | 0,850506 | 0,981381 | 0,980987 | 0,976357 | 0,984998 | 0,941125 | 0,951463 | 0,9851355 | 0,944121 |
| 25 vs. 50 | 0,789094 | 0,902814 | 0,510481 | 0,899384 | 0,895101 | 0,766555 | 0,878978 | 0,752973 | 0,842567 | 0,8870993 | 0,788689 |
|  |  |  |  |  |  |  |  |  |  |  |  |
|  | CPTAC GLOBAL TTEST | | |  |  |  |  |  |  |  |  |
| Comparison pair | Log2 | LoessF | LoessCyc | Rlr | RlrMA | RlrMACyc | Vsn | Quantile | Median | Progenesis | EigenMS |
| 0.25 vs. 0.74 | 0,575067 | 0,721067 | 0,703726 | 0,727062 | 0,716394 | 0,72516 | 0,708332 | 0,666501 | 0,652719 | 0,6200951 | 0,579905 |
| 0.25 vs. 2.2 | 0,786746 | 0,899711 | 0,89538 | 0,897564 | 0,897316 | 0,894674 | 0,909744 | 0,865909 | 0,845789 | 0,887531 | 0,782081 |
| 0.25 vs 6.7 | 0,950338 | 0,956889 | 0,973728 | 0,958411 | 0,959763 | 0,959256 | 0,97071 | 0,945228 | 0,930516 | 0,9642434 | 0,931826 |
| 0.74 vs 2.2 | 0,816908 | 0,802456 | 0,797062 | 0,795209 | 0,79368 | 0,793156 | 0,807045 | 0,801161 | 0,792874 | 0,8572464 | 0,815378 |
| 0.74 vs 6.7 | 0,91274 | 0,927827 | 0,937492 | 0,931961 | 0,933242 | 0,939567 | 0,932085 | 0,910126 | 0,89017 | 0,9281167 | 0,918279 |
| 2.2 vs 6.7 | 0,868488 | 0,898986 | 0,897168 | 0,902695 | 0,903973 | 0,917903 | 0,891238 | 0,8614 | 0,839062 | 0,8930926 | 0,868159 |
|  |  |  |  |  |  |  |  |  |  |  |  |
|  |  |  |  |  |  |  |  |  |  |  |  |
|  | SGSD GLOBAL TTEST | |  |  |  |  |  |  |  |  |  |
| Comparison pair | Log2 | LoessF | LoessCyc | Rlr | RlrMA | RlrMACyc | Vsn | Quantile | Median | Progenesis | EigenMS |
| 1 vs. 2 | 0,766393 | 0,799034 | 0,75826 | 0,805425 | 0,81089 | 0,801376 | 0,801864 | 0,812048 | 0,811963 | 0,746292 | 0,740578 |
| 1 vs .3 | 0,800527 | 0,935511 | 0,905865 | 0,935852 | 0,937854 | 0,929604 | 0,937268 | 0,929513 | 0,931849 | 0,8441222 | 0,802267 |
| 1 vs. 4 | 0,986485 | 0,933938 | 0,944428 | 0,933694 | 0,933938 | 0,951454 | 0,935597 | 0,924136 | 0,928376 | 0,9085187 | 0,952451 |
| 1 vs. 5 | 0,999415 | 0,999463 | 0,999413 | 0,999415 | 0,999415 | 0,99961 | 0,999415 | 0,998682 | 0,999415 | 0,9993169 | 0,998731 |
| 1 vs. 6 | 1 | 0,999756 | 0,999609 | 0,999805 | 0,999805 | 0,999805 | 0,999756 | 0,998583 | 0,999707 | 0,9995118 | 0,999853 |
| 1 vs. 7 | 1 | 1 | 1 | 1 | 1 | 1 | 1 | 0,999951 | 0,999951 | 1 | 0,999756 |
| 1 vs. 8 | 0,999707 | 0,999756 | 0,999902 | 0,999805 | 0,999805 | 0,999805 | 0,999805 | 0,999365 | 0,999805 | 0,9998046 | 0,999805 |
| 2 vs. 3 | 0,791154 | 0,846026 | 0,84824 | 0,852128 | 0,85208 | 0,849736 | 0,850029 | 0,840953 | 0,83968 | 0,8219586 | 0,781067 |
| 2 vs. 4 | 0,973409 | 0,951112 | 0,939492 | 0,955796 | 0,952625 | 0,960626 | 0,958285 | 0,95084 | 0,933255 | 0,9430621 | 0,951084 |
| 2 vs. 5 | 0,999317 | 0,998975 | 0,999071 | 0,999122 | 0,999122 | 0,999219 | 0,998975 | 0,997315 | 0,998878 | 0,999073 | 0,999512 |
| 2 vs. 6 | 0,999756 | 0,999658 | 0,999609 | 0,999756 | 0,999805 | 0,999707 | 0,999756 | 0,998095 | 0,999756 | 0,9994142 | 0,999902 |
| 2 vs. 7 | 0,999805 | 0,999951 | 0,999902 | 0,999951 | 0,999951 | 0,999951 | 0,999951 | 0,998828 | 1 | 0,999756 | 0,999805 |
| 2 vs. 8 | 0,999609 | 0,999316 | 0,999316 | 0,999414 | 0,999365 | 0,999365 | 0,999512 | 0,99917 | 0,999756 | 0,9994138 | 0,999658 |
| 3 vs. 4 | 0,899971 | 0,785686 | 0,806452 | 0,794376 | 0,78925 | 0,791154 | 0,791984 | 0,806549 | 0,739113 | 0,832357 | 0,854338 |
| 3 vs. 5 | 0,999756 | 0,999805 | 0,999804 | 0,999805 | 0,999805 | 0,999756 | 0,999805 | 0,999707 | 0,999561 | 0,9997071 | 0,999902 |
| 3 vs. 6 | 0,999902 | 0,999707 | 0,99956 | 0,999805 | 0,999756 | 0,999756 | 0,999756 | 0,997946 | 0,999756 | 0,9997071 | 0,999902 |
| 3 vs. 7 | 0,999902 | 0,999902 | 0,999853 | 0,999902 | 0,999902 | 0,999902 | 0,999902 | 0,999853 | 0,999902 | 0,9999024 | 1 |
| 3 vs. 8 | 0,999902 | 0,999511 | 0,999413 | 0,999462 | 0,999511 | 0,999462 | 0,999609 | 0,997801 | 0,999609 | 0,9996579 | 0,999804 |
| 4 vs. 5 | 0,999658 | 0,999317 | 0,999316 | 0,999171 | 0,999171 | 0,999268 | 0,999317 | 0,998193 | 0,999171 | 0,9991218 | 0,999951 |
| 4 vs. 6 | 1 | 0,999658 | 0,999218 | 0,999609 | 0,999561 | 0,999463 | 0,999658 | 0,998485 | 0,999658 | 0,9993654 | 1 |
| 4 vs. 7 | 0,999902 | 0,999902 | 0,999804 | 0,999951 | 0,999951 | 0,999951 | 1 | 0,998681 | 0,999951 | 0,9999024 | 0,999902 |
| 4 vs. 8 | 0,998779 | 0,999512 | 0,999462 | 0,99956 | 0,99956 | 0,99956 | 0,99956 | 0,997752 | 0,99956 | 0,999365 | 0,998925 |
| 5 vs. 6 | 0,868873 | 0,823618 | 0,821603 | 0,833968 | 0,832308 | 0,823081 | 0,834407 | 0,825509 | 0,834163 | 0,8276704 | 0,858587 |
| 5 vs. 7 | 0,981118 | 0,976922 | 0,977957 | 0,977996 | 0,977459 | 0,972629 | 0,975605 | 0,960606 | 0,971458 | 0,9708724 | 0,966266 |
| 5 vs. 8 | 0,920965 | 0,912466 | 0,898974 | 0,90973 | 0,913247 | 0,910219 | 0,913736 | 0,850821 | 0,910317 | 0,8858441 | 0,93049 |
| 6 vs. 7 | 0,764743 | 0,832845 | 0,838416 | 0,802871 | 0,825034 | 0,836507 | 0,839289 | 0,814994 | 0,863552 | 0,8389963 | 0,753908 |
| 6 vs. 8 | 0,762903 | 0,815005 | 0,787879 | 0,797165 | 0,811926 | 0,814907 | 0,808602 | 0,678935 | 0,79956 | 0,7843109 | 0,771701 |
| 7 vs. 8 | 0,678537 | 0,77711 | 0,814858 | 0,766462 | 0,778185 | 0,773593 | 0,774668 | 0,627149 | 0,750488 | 0,794451 | 0,699541 |
