# Supplementary material for: A systematic evaluation of normalization methods in quantitative label-free proteomics
Source: Brief Bioinform. 2016 Oct 2;19(1):1–11. doi: 10.1093/bib/bbw095 (PMC5862339; doi:10.1093/bib/bbw095)
Supplement: Supplementary Table2 [file bbw095_supplementary_table2.docx]

| Supplementary Table 2. **Areas under the ROC-curves (AUC) of the differential expression analysis using pairwise normalization and different test statistics.** Differential expression analyzed with ROTS and the common t-test. | | | | | | | | | | |
| --- | --- | --- | --- | --- | --- | --- | --- | --- | --- | --- |
|  |  |  |  |  |  |  |  |  |  |  |
|  | UPS1 PAIRWISE ROTS | | |  |  |  |  |  |  |  |
| Comparison pair | Log2 | LoessF | LoessCyc | Rlr | RlrMA | RlrMACyc | Vsn | Quantile | Median | EigenMS |
| 2 vs. 4 | 0,548168 | 0,88538 | 0,882961 | 0,881846 | 0,882421 | 0,844823 | 0,927714 | 0,866742 | 0,846176 | 0,858252 |
| 2 vs. 10 | 0,655862 | 0,989896 | 0,989241 | 0,986505 | 0,987166 | 0,987132 | 0,998457 | 0,986827 | 0,987946 | 0,672086 |
| 2 vs. 25 | 0,897395 | 0,995901 | 0,99665 | 0,995663 | 0,994986 | 0,995528 | 0,998425 | 0,995223 | 0,994952 | 0,87871 |
| 2 vs. 50 | 0,949589 | 0,993979 | 0,997613 | 0,994864 | 0,994575 | 0,995561 | 0,997874 | 0,996513 | 0,996003 | 0,902614 |
| 4 vs. 10 | 0,981603 | 0,96324 | 0,959146 | 0,957599 | 0,96241 | 0,95289 | 0,995375 | 0,958717 | 0,974658 | 0,981349 |
| 4 vs. 25 | 0,570693 | 0,987237 | 0,987344 | 0,984969 | 0,985291 | 0,983378 | 0,996818 | 0,976388 | 0,985934 | 0,565108 |
| 4 vs. 50 | 0,659931 | 0,982326 | 0,988479 | 0,979777 | 0,980219 | 0,979777 | 0,995463 | 0,974322 | 0,983482 | 0,762168 |
| 10 vs. 25 | 0,981296 | 0,974576 | 0,974804 | 0,978114 | 0,978063 | 0,980907 | 0,995159 | 0,953215 | 0,961797 | 0,97808 |
| 10 vs. 50 | 0,988979 | 0,968094 | 0,956298 | 0,97573 | 0,975339 | 0,97903 | 0,994966 | 0,951562 | 0,965355 | 0,962668 |
| 25 vs. 50 | 0,813275 | 0,76416 | 0,742258 | 0,863575 | 0,864169 | 0,863508 | 0,961855 | 0,670035 | 0,919861 | 0,825261 |
|  |  |  |  |  |  |  |  |  |  |  |
| Comparison pair | CPTAC PAIRWISE ROTS | | |  |  |  |  |  |  |  |
|  | Log2 | LoessF | LoessCyc | Rlr | RlrMA | RlrMACyc | Vsn | Quantile | Median | EigenMS |
| 0.25 vs. 0.74 | 0,66802 | 0,804959 | 0,794956 | 0,788045 | 0,790513 | 0,801894 | 0,729067 | 0,779011 | 0,719516 | 0,678088 |
| 0.25 vs. 2.2 | 0,868165 | 0,939622 | 0,933154 | 0,938466 | 0,937829 | 0,939582 | 0,92392 | 0,920014 | 0,911127 | 0,859118 |
| 0.25 vs 6.7 | 0,97314 | 0,966435 | 0,993407 | 0,972182 | 0,97342 | 0,970506 | 0,987508 | 0,972821 | 0,9644 | 0,972941 |
| 0.74 vs 2.2 | 0,835685 | 0,848677 | 0,828341 | 0,856608 | 0,849554 | 0,852582 | 0,832576 | 0,848199 | 0,847561 | 0,855173 |
| 0.74 vs 6.7 | 0,960192 | 0,965188 | 0,983672 | 0,967026 | 0,968345 | 0,957274 | 0,97482 | 0,955156 | 0,947882 | 0,965028 |
| 2.2 vs 6.7 | 0,936679 | 0,928154 | 0,954006 | 0,942163 | 0,942563 | 0,9382 | 0,967099 | 0,926433 | 0,90698 | 0,925672 |
|  |  |  |  |  |  |  |  |  |  |  |

|  | SGSD PAIRWISE ROTS | | |  |  |  |  |  |  |  |
| --- | --- | --- | --- | --- | --- | --- | --- | --- | --- | --- |
| Comparison pair | Log2 | LoessF | LoessCyc | Rlr | RlrMA | RlrMACyc | Vsn | Quantile | Median | EigenMS |
| 1 vs. 2 | 0,765271 | 0,817769 | 0,80645 | 0,808499 | 0,808499 | 0,80806 | 0,844067 | 0,816403 | 0,819379 | 0,753367 |
| 1 vs .3 | 0,788105 | 0,885051 | 0,881761 | 0,893052 | 0,896516 | 0,895638 | 0,911885 | 0,899053 | 0,89476 | 0,790496 |
| 1 vs. 4 | 0,972824 | 0,915203 | 0,903689 | 0,914861 | 0,915008 | 0,912324 | 0,920619 | 0,914861 | 0,908324 | 0,95887 |
| 1 vs. 5 | 0,998927 | 0,999512 | 0,999317 | 0,99961 | 0,999512 | 0,999463 | 0,99878 | 0,99961 | 0,998878 | 0,999122 |
| 1 vs. 6 | 1 | 1 | 1 | 1 | 1 | 0,999951 | 0,999902 | 1 | 0,999951 | 0,999756 |
| 1 vs. 7 | 1 | 1 | 1 | 1 | 1 | 1 | 1 | 1 | 1 | 1 |
| 1 vs. 8 | 0,999707 | 0,999902 | 0,999902 | 0,999902 | 0,999902 | 0,999902 | 0,999854 | 0,999756 | 0,999854 | 0,999805 |
| 2 vs. 3 | 0,753074 | 0,841335 | 0,841632 | 0,838554 | 0,837871 | 0,840798 | 0,850703 | 0,833724 | 0,833528 | 0,760246 |
| 2 vs. 4 | 0,977947 | 0,919887 | 0,913788 | 0,937549 | 0,937744 | 0,932523 | 0,922473 | 0,938378 | 0,912471 | 0,960529 |
| 2 vs. 5 | 0,999463 | 0,999561 | 0,999415 | 0,999561 | 0,999561 | 0,999415 | 0,99961 | 0,999512 | 0,999658 | 0,998927 |
| 2 vs. 6 | 1 | 1 | 1 | 1 | 1 | 1 | 1 | 1 | 1 | 1 |
| 2 vs. 7 | 1 | 0,999902 | 0,999951 | 0,999951 | 0,999951 | 0,999951 | 0,999902 | 0,999951 | 1 | 0,999902 |
| 2 vs. 8 | 0,999902 | 0,999805 | 0,999805 | 0,999854 | 0,999805 | 0,999854 | 0,999854 | 0,999805 | 0,999805 | 0,999707 |
| 3 vs. 4 | 0,884319 | 0,794155 | 0,787102 | 0,800205 | 0,800351 | 0,794936 | 0,819038 | 0,805133 | 0,717945 | 0,83187 |
| 3 vs. 5 | 0,999512 | 0,99961 | 0,999561 | 0,999756 | 0,999658 | 0,999707 | 0,999658 | 0,999463 | 0,999561 | 0,999707 |
| 3 vs. 6 | 1 | 1 | 1 | 1 | 1 | 1 | 1 | 1 | 0,999902 | 1 |
| 3 vs. 7 | 1 | 1 | 1 | 1 | 1 | 1 | 1 | 0,999854 | 1 | 1 |
| 3 vs. 8 | 0,999951 | 0,999707 | 0,999756 | 0,999707 | 0,999707 | 0,999707 | 0,999756 | 0,999756 | 0,999805 | 0,999854 |
| 4 vs. 5 | 0,999171 | 0,999024 | 0,999024 | 0,999024 | 0,999024 | 0,998878 | 0,998975 | 0,999024 | 0,998975 | 0,998195 |
| 4 vs. 6 | 1 | 1 | 1 | 1 | 1 | 1 | 1 | 1 | 1 | 1 |
| 4 vs. 7 | 1 | 1 | 1 | 1 | 1 | 1 | 1 | 0,999658 | 1 | 1 |
| 4 vs. 8 | 0,999317 | 0,999365 | 0,999316 | 0,999365 | 0,999414 | 0,999365 | 0,999365 | 0,999317 | 0,999561 | 0,999658 |
| 5 vs. 6 | 0,871438 | 0,861924 | 0,851982 | 0,86612 | 0,866023 | 0,851776 | 0,833724 | 0,842701 | 0,856509 | 0,867681 |
| 5 vs. 7 | 0,969262 | 0,972629 | 0,967701 | 0,97058 | 0,973263 | 0,958041 | 0,985802 | 0,955016 | 0,963603 | 0,964676 |
| 5 vs. 8 | 0,903827 | 0,912078 | 0,90382 | 0,907245 | 0,906219 | 0,893771 | 0,933119 | 0,846221 | 0,901923 | 0,905682 |
| 6 vs. 7 | 0,747024 | 0,782201 | 0,792033 | 0,763125 | 0,772883 | 0,767467 | 0,830406 | 0,706723 | 0,824649 | 0,742681 |
| 6 vs. 8 | 0,773726 | 0,772457 | 0,774536 | 0,747217 | 0,746924 | 0,74668 | 0,808875 | 0,617018 | 0,757811 | 0,750146 |
| 7 vs. 8 | 0,646163 | 0,667155 | 0,704914 | 0,661394 | 0,661394 | 0,677163 | 0,743214 | 0,57313 | 0,678871 | 0,651728 |

|  | UPS1 GLOBAL TTEST | |  |  |  |  |  |  |  |  |
| --- | --- | --- | --- | --- | --- | --- | --- | --- | --- | --- |
| Comparison pair | Log2 | LoessF | LoessCyc | Rlr | RlrMA | RlrMACyc | Vsn | Quantile | Median | EigenMS |
| 2 vs. 4 | 0,882461 | 0,675144 | 0,650074 | 0,676472 | 0,677058 | 0,655402 | 0,683058 | 0,674415 | 0,538088 | 0,559261 |
| 2 vs. 10 | 0,53062 | 0,987324 | 0,988792 | 0,983801 | 0,983473 | 0,988671 | 0,984802 | 0,971222 | 0,970261 | 0,574865 |
| 2 vs. 25 | 0,913512 | 0,995683 | 0,995182 | 0,995061 | 0,994802 | 0,994128 | 0,994646 | 0,979242 | 0,994007 | 0,869923 |
| 2 vs. 50 | 0,812195 | 0,994793 | 0,998443 | 0,993046 | 0,992285 | 0,996506 | 0,992562 | 0,9831 | 0,994586 | 0,642778 |
| 4 vs. 10 | 0,969955 | 0,887114 | 0,88466 | 0,875601 | 0,886754 | 0,87234 | 0,891815 | 0,847647 | 0,846242 | 0,981452 |
| 4 vs. 25 | 0,673257 | 0,917613 | 0,922744 | 0,881354 | 0,894438 | 0,888469 | 0,910498 | 0,836098 | 0,912414 | 0,54399 |
| 4 vs. 50 | 0,64219 | 0,909922 | 0,937147 | 0,862499 | 0,880141 | 0,89384 | 0,902412 | 0,815677 | 0,904075 | 0,497351 |
| 10 vs. 25 | 0,983071 | 0,961983 | 0,952586 | 0,967358 | 0,96758 | 0,97126 | 0,980486 | 0,935691 | 0,946697 | 0,956557 |
| 10 vs. 50 | 0,967065 | 0,970151 | 0,918014 | 0,9737 | 0,973769 | 0,982632 | 0,98493 | 0,942925 | 0,951463 | 0,929432 |
| 25 vs. 50 | 0,789094 | 0,8131 | 0,825982 | 0,848044 | 0,846491 | 0,856302 | 0,888464 | 0,755038 | 0,842567 | 0,776297 |
|  |  |  |  |  |  |  |  |  |  |  |
|  | CPTAC GLOBAL TTEST | | |  |  |  |  |  |  |  |
| Comparison pair | Log2 | LoessF | LoessCyc | Rlr | RlrMA | RlrMACyc | Vsn | Quantile | Median | EigenMS |
| 0.25 vs. 0.74 | 0,575067 | 0,71379 | 0,713045 | 0,707091 | 0,70829 | 0,725822 | 0,70275 | 0,666128 | 0,652719 | 0,554311 |
| 0.25 vs. 2.2 | 0,786746 | 0,89616 | 0,887737 | 0,896201 | 0,895747 | 0,898018 | 0,901569 | 0,866033 | 0,845789 | 0,817506 |
| 0.25 vs 6.7 | 0,950338 | 0,954396 | 0,976289 | 0,957227 | 0,95896 | 0,960989 | 0,967202 | 0,946667 | 0,930516 | 0,927557 |
| 0.74 vs 2.2 | 0,816908 | 0,784944 | 0,785668 | 0,793559 | 0,791224 | 0,789291 | 0,793478 | 0,800798 | 0,792874 | 0,820089 |
| 0.74 vs 6.7 | 0,91274 | 0,928282 | 0,947173 | 0,932829 | 0,936053 | 0,940972 | 0,932829 | 0,911244 | 0,89017 | 0,91307 |
| 2.2 vs 6.7 | 0,868488 | 0,880646 | 0,902489 | 0,904385 | 0,904921 | 0,912298 | 0,88893 | 0,860822 | 0,839062 | 0,866963 |
|  |  |  |  |  |  |  |  |  |  |  |
|  |  |  |  |  |  |  |  |  |  |  |

|  | SGSD GLOBAL TTEST | |  |  |  |  |  |  |  |  |
| --- | --- | --- | --- | --- | --- | --- | --- | --- | --- | --- |
| Comparison pair | Log2 | LoessF | LoessCyc | Rlr | RlrMA | RlrMACyc | Vsn | Quantile | Median | EigenMS |
| 1 vs. 2 | 0,766393 | 0,800595 | 0,79396 | 0,807767 | 0,80767 | 0,805816 | 0,804645 | 0,80951 | 0,811963 | 0,742438 |
| 1 vs .3 | 0,800527 | 0,936243 | 0,933607 | 0,938244 | 0,937707 | 0,937317 | 0,936389 | 0,930295 | 0,931849 | 0,7996 |
| 1 vs. 4 | 0,986485 | 0,926717 | 0,92418 | 0,936817 | 0,937256 | 0,932328 | 0,935158 | 0,924868 | 0,928376 | 0,973165 |
| 1 vs. 5 | 0,999415 | 0,999463 | 0,999415 | 0,999463 | 0,999463 | 0,999463 | 0,999512 | 0,998731 | 0,999415 | 0,998536 |
| 1 vs. 6 | 1 | 0,999756 | 0,999707 | 0,999805 | 0,999805 | 0,999805 | 0,999756 | 0,998632 | 0,999707 | 0,999219 |
| 1 vs. 7 | 1 | 1 | 1 | 1 | 1 | 1 | 1 | 0,999951 | 0,999951 | 1 |
| 1 vs. 8 | 0,999707 | 0,999805 | 0,999805 | 0,999805 | 0,999805 | 0,999805 | 0,999805 | 0,999609 | 0,999805 | 0,999707 |
| 2 vs. 3 | 0,791154 | 0,850469 | 0,852324 | 0,852763 | 0,852275 | 0,852324 | 0,854667 | 0,838804 | 0,83968 | 0,773823 |
| 2 vs. 4 | 0,973409 | 0,947502 | 0,942233 | 0,958528 | 0,958577 | 0,953308 | 0,94555 | 0,949522 | 0,933255 | 0,940818 |
| 2 vs. 5 | 0,999317 | 0,998975 | 0,999073 | 0,999073 | 0,999122 | 0,999073 | 0,998975 | 0,997413 | 0,998878 | 0,998048 |
| 2 vs. 6 | 0,999756 | 0,999756 | 0,999805 | 0,999756 | 0,999756 | 0,999756 | 0,999756 | 0,998193 | 0,999756 | 0,999756 |
| 2 vs. 7 | 0,999805 | 0,999951 | 0,999951 | 0,999951 | 0,999951 | 0,999951 | 1 | 0,998828 | 1 | 0,99961 |
| 2 vs. 8 | 0,999609 | 0,999414 | 0,999365 | 0,999414 | 0,999365 | 0,999463 | 0,99956 | 0,999267 | 0,999756 | 0,99917 |
| 3 vs. 4 | 0,899971 | 0,788469 | 0,784319 | 0,795157 | 0,795401 | 0,792423 | 0,796866 | 0,803763 | 0,739113 | 0,838996 |
| 3 vs. 5 | 0,999756 | 0,999805 | 0,999805 | 0,999805 | 0,999805 | 0,999756 | 0,999854 | 0,999707 | 0,999561 | 0,99917 |
| 3 vs. 6 | 0,999902 | 0,999707 | 0,999707 | 0,999805 | 0,999756 | 0,999756 | 0,999805 | 0,997946 | 0,999756 | 0,999707 |
| 3 vs. 7 | 0,999902 | 0,999902 | 0,999902 | 0,999854 | 0,999854 | 0,999902 | 0,999902 | 0,999853 | 0,999902 | 1 |
| 3 vs. 8 | 0,999902 | 0,999511 | 0,999365 | 0,999511 | 0,999413 | 0,999462 | 0,999658 | 0,997996 | 0,999609 | 0,997752 |
| 4 vs. 5 | 0,999658 | 0,999317 | 0,999317 | 0,999171 | 0,999171 | 0,999171 | 0,999073 | 0,998144 | 0,999171 | 0,994779 |
| 4 vs. 6 | 1 | 0,999561 | 0,999512 | 0,999561 | 0,999561 | 0,999463 | 0,999707 | 0,998485 | 0,999658 | 0,997413 |
| 4 vs. 7 | 0,999902 | 0,999854 | 0,999951 | 0,999951 | 0,999951 | 0,999951 | 1 | 0,998681 | 0,999951 | 0,999805 |
| 4 vs. 8 | 0,998779 | 0,999512 | 0,999512 | 0,999512 | 0,99956 | 0,999609 | 0,999512 | 0,998192 | 0,99956 | 0,998339 |
| 5 vs. 6 | 0,868873 | 0,840217 | 0,828744 | 0,841632 | 0,841779 | 0,827817 | 0,830307 | 0,822379 | 0,834163 | 0,857987 |
| 5 vs. 7 | 0,981118 | 0,978191 | 0,977703 | 0,979557 | 0,979557 | 0,976483 | 0,977117 | 0,961877 | 0,971458 | 0,959797 |
| 5 vs. 8 | 0,920965 | 0,916081 | 0,91061 | 0,912954 | 0,913443 | 0,902159 | 0,912612 | 0,859662 | 0,910317 | 0,885502 |
| 6 vs. 7 | 0,764743 | 0,831039 | 0,842755 | 0,808485 | 0,808924 | 0,80331 | 0,8347 | 0,809174 | 0,863552 | 0,741506 |
| 6 vs. 8 | 0,762903 | 0,817253 | 0,818475 | 0,796041 | 0,795259 | 0,783724 | 0,807331 | 0,680254 | 0,79956 | 0,74956 |
| 7 vs. 8 | 0,678537 | 0,784828 | 0,801338 | 0,764166 | 0,763921 | 0,762114 | 0,775401 | 0,630178 | 0,750488 | 0,659486 |
